# Underuse of long-term routine hospital follow-up care in patients with a history of breast cancer?

**DOI:** 10.1186/1471-2407-11-279

**Published:** 2011-06-28

**Authors:** Wenli Lu, Liesbeth Jansen, Michael Schaapveld, Peter C Baas, Theo Wiggers, Geertruida H De Bock

**Affiliations:** 1 Department of Epidemiology, University Medical Center Groningen, University of Groningen, The Netherlands; 2 Department of Health Statistics, School of Public Health, Tianjin Medical University, China; 3 Department of Epidemiology, Tianjin Medical University Cancer Institute and Hospital, China; 4 Department of Surgery, University Medical Center Groningen, University of Groningen, The Netherlands; 5 Comprehensive Cancer Centre North-Netherlands, The Netherlands; 6 Department of Surgery, Martiniziekenhuis, The Netherlands

**Keywords:** Breast neoplasm, Utilization, Follow-up, Mammography

## Abstract

**Background:**

After primary treatment for breast cancer, patients are recommended to use hospital follow-up care routinely. Long-term data on the utilization of this follow-up care are relatively rare.

**Methods:**

Information regarding the utilization of routine hospital follow-up care was retrieved from hospital documents of 662 patients treated for breast cancer. Utilization of hospital follow-up care was defined as the use of follow-up care according to the guidelines in that period of time. Determinants of hospital follow up care were evaluated with multivariate analysis by generalized estimating equations (GEE).

**Results:**

The median follow-up time was 9.0 (0.3-18.1) years. At fifth and tenth year after diagnosis, 16.1% and 33.5% of the patients had less follow-up visits than recommended in the national guideline, and 33.1% and 40.4% had less frequent mammography than recommended. Less frequent mammography was found in older patients (age > 70; OR: 2.10; 95%CI: 1.62-2.74), patients with comorbidity (OR: 1.26; 95%CI: 1.05-1.52) and patients using hormonal therapy (OR: 1.51; 95%CI: 1.01-2.25).

**Conclusions:**

Most patients with a history of breast cancer use hospital follow-up care according to the guidelines. In older patients, patients with comorbidity and patients receiving hormonal therapy yearly mammography is performed much less than recommended.

## Background

Breast cancer is the commonest incident form of malignancy among women in Europe[1]. Advances in the early diagnosis and treatment of breast cancer increase the number of breast cancer survivors[2]. Nowadays, it is estimated that more than 80% of the patients with a history of breast cancer will survive longer than 5 years and more than 60% of these patients will survive longer than 10 years[3,4]. After primary treatment, these patients are offered routine hospital follow-up. One of the main aims is to early detect loco-regional recurrences and second primary tumours. Approximately 40% of the isolated locoregional recurrences are diagnosed during routine visits and routine tests in asymptomatic patients treated for early-stage invasive breast cancer [5]. In a recently published meta-analysis it was shown that the detection of isolated loco-regional or contra-lateral breast cancer recurrences in patients without symptoms has beneficial impact on survival of breast cancer patients when compared to late symptomatic detection, indicating that regular hospital follow-up might be beneficial for some patients [6]. Although recommendations vary to some extent with regards to timing, routine hospital follow-up visits include history taking, physical examination and mammography are part of the schedule. Health care utilization after primary treatment for women with a history of breast cancer represents a major public health issue with increasing importance [7,8].

Routine hospital follow-up care is a major portion of health care use of breast cancer survivors [8]. There is a limited number of studies examining the health care use of long-term follow up care of breast cancer survivors including hospital visits (history taking and clinical examination) and mammography[9,10]. Data on utilization of long-term follow-up care beyond five years of breast cancer patients are relatively rare as well as on factors associated with the utilization of long-term follow-up care [7,11]. The aim of the current study was to assess the use of long-term routine hospital follow-up care, to describe the reasons of stopping this follow-up care, and to assess the determinants of the use of this routine hospital follow-up care. For that we derived information from hospital documents of 662 patients treated for breast cancer.

## Methods

### Settings and subjects

The regional cancer registry of the Comprehensive Cancer Centre North-Netherlands (CCCN, merged into the Comprehensive Cancer Centre Northeast-Netherlands in 2009) was used to select the patients who were diagnosed with breast cancer from January 1989 to January 2003. This cancer registry contains data on diagnosis, stage, and treatment actively abstracted from the patient's medical records in all hospitals within the CCCN catchment area using the registration and coding manual of the Dutch Association of Comprehensive Cancer Centres. Passive follow-up of vital status through municipal population registries is conducted and the vital status of patients is updated annually.

**Figure 1:**
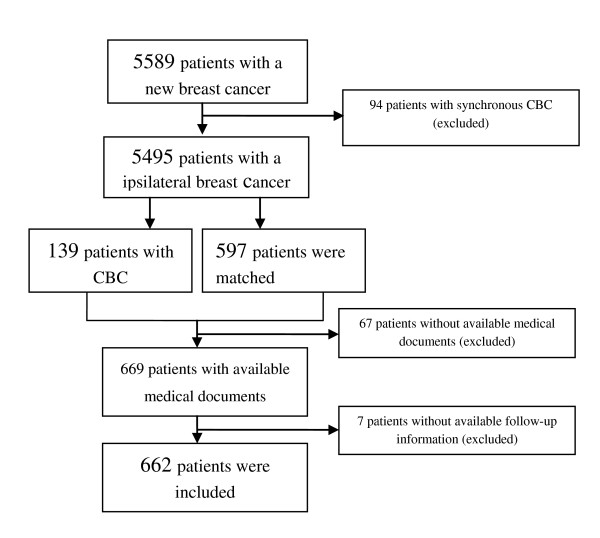
Graphical illustration of the sampling of study cohort

Of these 5,589 women a total of 139 patients developed contra lateral breast cancer (CBC) after six months since the first tumour and those contra lateral breast cancers were registered in the database. Because follow-up data was not available in the cancer registry, and we were not able to retrieve the follow-up data from all 5,495 patients from the hospital files. We decided to over-sample the women with contralateral breast cancer and sampled a subset of women without contralateral breast cancer. This cohort is representative of breast cancer survivors at a greater risk of secondary breast cancer supposed to have a better utilization of follow-up procedures. For each woman with CBC, four to five patients without evidence of CBC - matched with hospital of diagnosis, age at first primary tumour, and duration of follow-up - were selected randomly in this cohort. Based on this a cohort of 736 patients was constructed including all women with a CBC. Medical documents were unavailable for 67 (9.1%) patients, and for seven (1.0%) patients there was no information available on the follow-up. Finally, there were 662 (89.9%) patients in our study with information concerning at least one year follow-up (See Figure 1).

### Data abstraction

For all patients in our study, the follow-up information was retrieved actively from hospital documents, including date and content of visits, date of mammography. In addition, data on family history and comorbidity were retrieved for the patients in our study, as these data were not included in the cancer registry. Comorbidity was assessed with the use of the slightly modified Charlson comorbidity index [12,13]. Per visit, it was retrieved whether patients came to the hospital with or without symptoms. In addition information on the following events was retrieved: the incidence of any loco-regional recurrences, contra-lateral breast cancers, distant metastasis or death. The time of ending hospital follow-up and the reason was retrieved as well. The information on whether the patient went to the Dutch National Breast Cancer Screening Program was derived from linkage between the CCCN and the Dutch National Breast Cancer Screening Program.

### Follow-up guidelines in the study period

After surgical treatment for breast cancer, follow-up was offered four times a year in the first two years. After two years it was offered two times a year and after five years it was offered once a year. Follow-up included history and physical examination. Mammography was offered once in two years (guidelines published in 1987). In 1994 the preferred frequency of mammography was changed from once in two years to yearly. The duration for follow-up was not specified. In the Northern part of the Netherlands, in 1999 follow-up was stratified for age groups. If the patient was under the age of 70 at the start of surgical treatment, hospital follow-up was offered for a period of 10 years, and after that period patients were referred back to their General Practitioner. If the patient was 70 or older at the start of surgical treatment, hospital follow-up was offered for a period of 5 years, and after that period patients were referred back to their GP. In 2003 the age cut-off was changed from 70 to 65 and the option to refer patients to the Dutch National Breast Cancer Screening Program was added after the ending of the hospital follow-up. That time breast cancer screening in the Dutch National Breast Cancer Screening Program was offered to all women from 50 till 75 years of age.

### Definitions

For each patient, the time for routine hospital follow-up was assessed. The routine hospital follow-up time was considered to be ended if the patient was diagnosed with a loco-regional recurrence, a contra-lateral recurrence, a distant metastasis or death. In addition, the routine hospital follow-up time was considered to be ended if there was a note in the files about ending the follow-up which was not followed by any other follow-up visit. When there is no record of ending the routine hospital follow-up, the retrieval date was considered as the last date of follow-up.

If a visit or a diagnostic mammography was related to a patient that presented with symptoms, the related visit and the related diagnostic mammogram were not considered as being part of routine hospital follow-up and these visits were excluded from the analysis.

The utilization of hospital follow-up for every follow-up person-year was evaluated after 1994 when the frequency of follow-up was recommended to be given once a year. This hospital follow-up was evaluated according to the guidelines of follow-up of patient with a breast cancer. Underuse of hospital follow-up was defined as there was no hospital follow-up in patients who supposed to have hospital follow-up during that period according to the guidelines. The underuse of mammography was defined as having no mammogram in a period of at least 14 months in women who were followed-up in hospital. Women whose time between two mammograms was less than 10 months were first identified and then evaluated whether the second mammogram was appropriate according to the guidelines, if not, it would be considered as an overuse of annually mammography.

### Statistical analysis

Determinants potentially related to the underuse of hospital follow-up care, mammography were evaluated in a multivariate model by generalized estimating equations (GEE) which accounted for the dependent outcomes within breast cancer survivors. Odds ratios (OR) were calculated to reflect the potential associations between determinants and outcomes. Outcomes considered were whether patients underused hospital visits or yearly mammography after primary treatment (yes or no). Determinant potentially related were age at primary diagnosis, year at diagnosis, year after primary treatment, whether the first tumour was detected during national breast cancer screening program, family history, comorbidity, characteristics of first tumour and treatment for first tumour. The periods of each constant guideline were taken into accounted as covariate. Only results from multivariate analyses are presented.

## Results

### Patients' characteristics

Of the 662 women included in this study, 197 (29.8%) patients were younger than 50 years of age at diagnosis of the first tumour and the median age was 57.7 (26-93). Family history of breast cancer was present in 150 (22.7%) patients and comorbidity was present in 276 (41.7%) patients. 423 (63.9%) patients had mastectomy and 214(32.3%) had had breast conserving therapy for their first tumour. 20.1% of the patients received chemotherapy, and 138(20.8%) of the patients received hormonal therapy. The median follow-up time was 9.0 years (0.3-18.1) (See Table 1).

**Table 1:** Characteristics of patients and their first breast cancer n (%)

Characteristics	N	%
Age at diagnosis [median(range) yrs]	57.7	26-93
Age group at diagnosis		
<50	197	29.8
50-59	172	26.0
60-69	163	24.6
70+	130	19.6
Year of diagnosis		
1989-1993	313	47.3
1994-2002	349	52.7
Detecting first tumour by Screening programme		
No	335	72.0
Yes	130	28.0
No applicable	197	
Co morbidity		
No	386	58.3
Yes	276	41.7
Pathologic T stage		
pTis/T1	376	57.7
pT2-3	276	42.3
Unknown	10	
Pathologic N stage		
N0	435	62.2
N1-3	212	37.8
Unknown	10	
Surgery		
BCT	214	33.6
Mastectomy	423	66.4
Unknown	25	
Radiation therapy		
Yes	284	42.9
No	378	57.1
Chemotherapy		
Yes	98	20.8
No	564	79.2
Hormonal therapy		
Yes	138	79.2
No	524	20.8

### Ending of hospital follow-up

Of the 662 women, routine hospital follow-up was documented to be ended for 247(37.3%) among which 90 (36.4%) patients the time of stopping their hospital follow-up was earlier than it would have been based on the guidelines. Out of these 90 patients, 38 (42.2%) patients followed the doctor's advice to stop being hospital follow-up. 18 (20.0%) patients themselves decided to stop hospital follow-up, 9 (10.0%) patients stopped routine hospital follow-up because of comorbidity and 25 (27.8%) patients were transferred to the National Breast Cancer Screening Program sooner than was recommended by guidelines. 178 (72.1%) out of 247 patients were younger than 75 years of age at the time of ending their routine hospital follow-up. Of these, 156 (63.2%) patients were between 50 and 75 years and, 48 (30.7%) patients were transferred to the National Breast Cancer Screening Program. For 90 (36.4%) patients the time of stopping their hospital follow-up was earlier than it would have been based on the guidelines.

### Utilization of hospital follow-up visits

The long-term routine hospital follow-up visits decreased over time gradually. 2.4%, 16.1% and 33.5% patients had less follow-up visits than recommended in the national guideline at the first, fifth and tenth year after treatment (see Table 2). The total number of registered contacts was 10,553. Patients had an average of five visits in the first year, three times in the second year, twice in the third to fifth year and once in the years after. During their hospital follow-up, 109 (16.5%) patients were found both to underuse and to overuse hospital follow-up visits, 221 (33.4%) patients were found to underuse hospital follow-up visits, 99 (15.0%) patients were found to overuse hospital follow-up visits and 233 (35.2%) patients were compliant with the recommended frequency of hospital follow-up visits.

**Table 2:** Utilization of breast cancer follow-up care in breast cancer survivors

Time after treatment (years)	Disease free Patients*	Patients used hospital follow-up visit		Patients used yearly mammography	
	
	n	n	%	n	%
1	662	639	96.5	282	44.1
2	634	587	92.6	412	70.2
3	613	548	89.4	368	67.2
4	590	503	85.3	331	65.8
5	570	478	83.9	320	66.9
6	538	447	83.1	277	62.0
7	516	400	77.5	246	61.5
8	470	340	72.3	208	61.2
9	425	300	70.6	194	57.1
10	361	240	66.5	143	59.6

* Patients who have a loco-regional recurrence or contra-lateral breast cancer were excluded. Patients who have distant metastasis were excluded.

In the multivariate model, as compared to patients aged 50-69 years, women who had breast cancer diagnosed after 70 years of age were less likely to underuse hospital follow up (OR: 0.64, 95%-CI: 0.44-0.89). Women who receive radiotherapy for the first tumour were less likely to underuse the hospital follow-up (OR: 0.53, 95%-CI: 0.29-0.95). Compared to the first five years after primary treatment, women were more likely to underuse the hospital follow up in the sixth to the tenth year from the primary treatment of first tumour (OR: 2.86, 95%-CI: 2.26-3.62).

### Utilization of mammography

Only 44.1% patients had a mammogram with 14 months after completion of primary treatment. 33.1% and 40.4% of the patients had less mammogram in the fifth and tenth year after primary treatment, respectively (see Table 2). During their hospital follow-up, 112 (17.0%) patients were found both to underuse and to overuse mammography, 506 (76.7%) patients were found to underuse mammography, 12 (1.8) patients were found to overuse mammography and 30 (4.5%) patients were compliant with the recommended frequency of mammography.

In the multivariate model evaluating the potential factors associated with the underuse of mammography, older age (age > 70; OR: 2.10; 95%CI: 1.62-2.74), patients with co-morbidities (OR: 1.26; 95%CI: 1.05-1.52) and hormonal therapy (OR: 1.51; 95%CI: 1.01-2.25). Patient with their first tumour diagnosed after 1994 (OR: 0.74; 95%CI: 0.60-0.92), patients with positive lymph node at the first tumour (OR: 0.65; 95%CI: 0.42-0.98) and patients who received radiotherapy (OR: 0.73; 95%CI: 0.55-0.98) were less likely to underuse yearly mammography. After five years of follow-up, patients were more likely to underuse yearly mammography according to the guidelines (for 6-10 year was OR: 1.74; 95%CI: 1.48-2.06 and OR for 10+ year was 8.21, 95%-CI: 6.05-11.14, see Table 3).

**Table 3:** Predictive factor of underuse of follow-up care in breast cancer survivors (multivariate results)

Variable		OR	95% CI
Underuse of hospital follow-up			
Age at diagnosis	<50	1.42	0.95-2.11
	50-69	1	
	70-	0.64	0.42-0.96
Year at diagnosis	1989-1993	1	
	1994-2002	0.63	0.44-0.89
Year after primary treatment	1-5	1	
	6-10	2.86	2.26-3.62
	10+	-	-
Radiation therapy	No	1	
	Yes	0.53	0.29-0.95

Underuse of yearly of mammography			

Age at diagnosis	<50	1.29	1.00-1.67
	50-69	1	
	70-	2.10	1.62-2.74
Year at diagnosis	1989-1993	1	
	1994-2002	0.74	0.60-0.92
Year after primary treatment	1-5	1	
	6-10	1.74	1.48-2.06
	10+	8.21	6.05-11.14
Co morbidity	No	1	
	Yes	1.26	1.05-1.52
pN stage	N0	1	
	N1/N2/N3	0.65	0.42-0.98
Radiation therapy	No	1	
	Yes	0.73	0.55-0.98
Hormonal therapy	No	1	
	Yes	1.51	1.01-2.25

## Discussion

This study evaluated utilization of hospital follow-up in a cohort of 662 patients with a median follow-up time of 9.0 years (range: 0.1-18.2). At fifth and tenth year after diagnosis, 16.1% and 33.5% of the patients were under using hospital follow-up care, respectively, where of the patients in hospital follow-up, 33.1% and 40.4% of the patients were under using mammography, respectively. Underuse of yearly mammography was found in older patients (age > 70; OR: 2.10; 95%CI: 1.62-2.74), patients with comorbidity (OR: 1.26; 95%CI: 1.05-1.52) and patients with hormonal therapy (OR: 1.51; 95%CI: 1.01-2.25).

Routine hospital follow-up care was ended for 37.3% (n = 247) recurrence-free patients. For one-third of these patients (n = 90; 36.4%) the time of stopping their hospital follow-up was earlier than it should have been based on the guidelines. According to the guidelines, women with a history of breast cancer should be advised to be followed-up until age of 75 either in hospital or being transferred to the National Breast Cancer Screening Program. Only one-third of the patients who were at age of between 50 and 75 at the time of ending follow-up were indeed transferred to the National Breast Cancer Screening Program. Patients between the age of 50 and 75, ending their routine hospital follow-up program should be well actively referred to the National Breast Cancer Screening Program. Recently this was added to the Dutch national guideline for breast cancer screening [http://www.oncoline.nl/index.php?pagina=/richtlijn/item/pagina.php&richtlijn_id=593].

Patients who received radiation therapy for their primary tumours and women with positive lymph node at the first tumour were less likely to underuse the routine hospital follow-up. This might be a reflection of more concern about the risk of breast cancer recurrences and the more consequent recall policy of the radiation oncologist.

Compared to first five years after primary treatment, women were more likely underuse the hospital follow up in the sixth to the tenth year from the primary treatment of first tumour (OR: 2.86, 95%-CI: 2.26-3.62). This finding is consistent with another research and the decreasing concern about the risk of breast cancer over time may be a possible explanation for declining routine hospital follow-up visits [14].

In this study, the utilization of mammography was evaluated in women who were followed up in hospital. For patients who stopped their hospital follow-up, the utilization of mammography was not evaluated. Mammography is the only recommended imaging test for the routine follow-up of women with a history of breast cancer and is recommended by most guidelines [15]. Underutilization of mammography was still found in 30-40% patients during the routine hospital follow-up phase in this study. Several studies reported that elderly patients are less likely to receive routine mammography [14,16] which is also the case in this study, patients over 70 had a lower chance to receive yearly mammography. An explanation might be that among elderly, there is a decreasing concern about the risk of breast cancer recurrences [16]. Lower use among the elderly could be related to competing medical needs and/or perceived diminishing benefit from regular mammography with advancing age also. Patients who had comorbidity and patient who received radiation therapy had fewer mammograms than advised. These findings may reflect potentially appropriate decisions by patients or their physicians for less intensive care as a result of competing comorbid illness causing that follow-up mammography became a lower priority in these patients[14,16,17]. The finding that the patients who received hormonal therapy had less frequent mammography than advised might be a reflection of the awareness of the decreasing efficiency of mammography in follow-up after recalculating the incidence of local recurrences which is reduced by hormonal therapy. The patients with a first tumour diagnosed after 1994 were more likely to have yearly mammography probably due to the increasing awareness of the benefit of mammography and the publications of guideline on follow-up of breast cancer which addressed the importance of mammography[18-20].

As well as the use of routine hospital follow-up, the use of yearly mammography decreased gradually over years. It may be an effective and safe choice to transfer women with a history of breast cancer to their general practitioner to keep the continuity of care at an appropriate time[21]. This may reflect tailored follow-up schedules, where the estimated risk of recurrence influences the choice to adhere to the follow-up schedule as in the national guidelines or to decrease the number of follow-up visits and examinations. Because the recurrence rate in the study population cannot be assessed reliable from the present data, this hypothesis cannot be tested.

Caution is needed when generalizing study results and conclusions to other population and settings. Our study cohort consists of patients with metachronous contra-lateral breast cancers and random group stratified on hospital of diagnosis, age and duration of follow-up. This cohort is representative of breast cancer survivors at a greater risk of secondary breast cancer who are expected to be under close surveillance. The other limitation of present study is we only reviewed the utilization of hospital follow-up. It is still unclear whether patients who stop their hospital follow-up were followed up in the National Breast Screening Program or in general practice routinely or not. From other research it became clear that patients who continued to see cancer specialists after their initial cancer treatment were more likely than other patients to undergo routine follow-up mammography [22,23]. The utilization of follow-up care elsewhere such as the National Breast Screening Program or in general practice for long term survivors deserves further research. The consequence of underuse of hospital follow-up is not clear due to the lack of conclusive evidence in the literature about the benefit of regular hospital follow-up visits, either in terms of early detection of recurrences or in relation to psychological well-being.

## Conclusions

Most patients with a history of breast cancer use hospital follow-up care according to the guidelines. In older patients, patients with comorbidity and patients receiving hormonal therapy we should be careful to ensure the use of yearly mammography.

## Abbreviations

GEE: Generalized estimating equations; OR: Odds ratio; CCCN: Comprehensive Cancer Centre North-Netherlands; CBC: Contra lateral breast cancer

## Competing interests

The authors declare that they have no competing interests.

## Authors' contributions

WL, MS, LJ and GHdB have made substantial contributions to the conception and design of the study, and to the analysis and interpretation of data. WL and GB have been involved in drafting the manuscript. PB and TW has been involved in revising the manuscript critically.

All authors have participated sufficiently in the work to take public responsibility for appropriate portions of the content.

All authors read and approved the final manuscript.

## Pre-publication history

The pre-publication history for this paper can be accessed here:

http://www.biomedcentral.com/1471-2407/11/279/prepub
